# Multidisciplinary Management of Common Bile Duct Metastasis from Rectal Cancer: A Case Report of Long-Term Disease Control and Literature Review

**DOI:** 10.70352/scrj.cr.26-0214

**Published:** 2026-09-11

**Authors:** Yudai Kikkawa, Masatoshi Kochi, Yuki Tokumoto, Yuta Hiura, Makoto Shinohara, Megumi Yamaguchi, Yusuke Sumi, Michinori Hamaoka, Ryuichi Hotta, Takuya Hattori, Kazuhiro Toyota

**Affiliations:** Department of Gastroenterological Surgery, National Hospital Organization (NHO) Higashihiroshima Medical Center, Higashihiroshima, Hiroshima, Japan

**Keywords:** common bile duct metastasis, colorectal cancer, extrahepatic bile duct metastasis

## Abstract

**INTRODUCTION:**

Extrahepatic bile duct (EHBD) metastasis from colorectal cancer is extremely rare and remains a diagnostic and therapeutic challenge. Its clinical and radiological features often mimic primary cholangiocarcinoma and, because of the limited number of reported cases, an optimal management strategy has not yet been established.

**CASE PRESENTATION:**

A 46-year-old man underwent curative resection for rectal cancer with synchronous liver metastasis, followed by laparoscopic hepatectomy. Eight months after hepatic resection, metachronous recurrence involving the common bile duct was detected, together with local recurrence. Histopathological examination and immunohistochemical analysis of the bile duct lesion revealed a cytokeratin 7-negative, cytokeratin 20-positive, and caudal-type homeobox 2-positive profile, confirming metastasis from rectal adenocarcinoma. Because of unresectable recurrent disease, systemic chemotherapy was initiated. The patient subsequently developed obstructive jaundice due to bile duct metastasis and underwent endoscopic biliary stent placement. Although multiple stent exchanges were required because of stent occlusion and migration, biliary drainage was successfully maintained, allowing continuation of chemotherapy. The patient survived for 24 months following diagnosis of EHBD metastasis with preserved QOL.

**CONCLUSIONS:**

EHBD metastasis from colorectal cancer is a rare condition with no established treatment consensus. This case highlights that, even in unresectable disease, careful long-term management combining systemic chemotherapy with endoscopic biliary stenting may contribute to sustained biliary control and prolonged survival. Individualized treatment planning and close follow-up are essential, particularly for managing stent-related complications.

## INTRODUCTION

In a large multicenter cohort, the most common first recurrence sites within 5 years after curative resection for stage II/III colorectal cancer were the liver and lung, followed by peritoneal and local recurrences.^1)^

Metastasis to the Extrahepatic bile duct (EHBD) is an extremely rare manifestation of colorectal cancer, and the mechanism of its development remains unclear owing to its rarity. Its clinical and radiological presentations are often indistinguishable from primary cholangiocarcinoma.^2)^

We report a rare case of metachronous recurrence of rectal cancer involving the common bile duct after curative resection of the primary rectal cancer and liver metastasis. The patient achieved remarkably long survival without surgical resection through multidisciplinary management, including systemic chemotherapy and palliative biliary stenting.

## CASE PRESENTATION

A 46-year-old man presented with nausea and constipation and was admitted to our hospital for further evaluation. He had no significant past medical history, was not taking any medications, and had no family history of cancer.

**Fig. 1:**
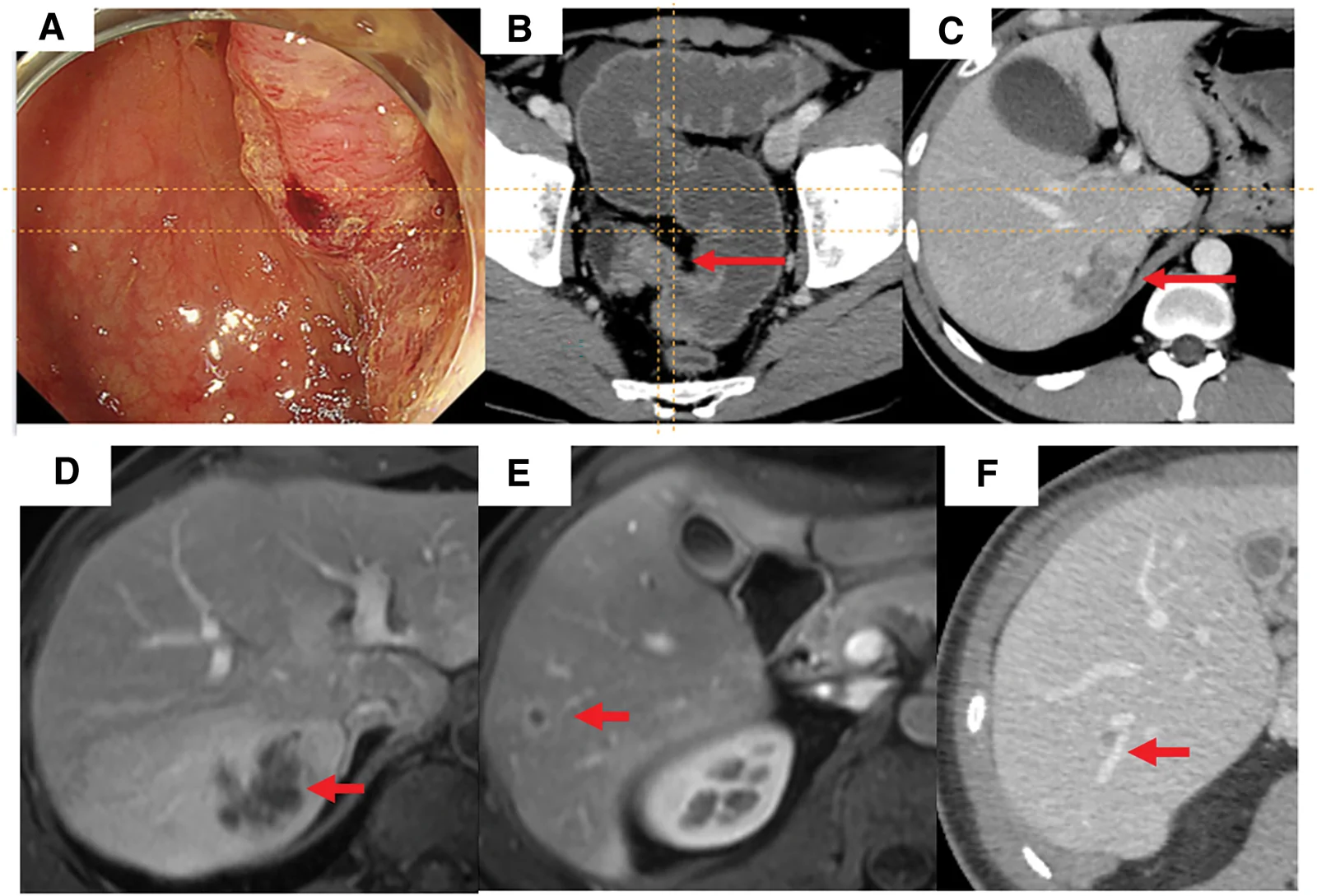
(**A**) Colonoscopy depicting a circumferential tumor. (**B**) Contrast-enhanced CT showing wall thickening of the rectum with proximal bowel dilatation. (**C**) Contrast-enhanced CT showing a 35-mm metastatic lesion in segment VII of the liver. (**D**) Gadoxetic acid-enhanced MRI illustrating a 35-mm metastatic lesion in segment VII of the liver. (**E**) Gadoxetic acid-enhanced MRI of a ring-enhancing metastatic nodule in segment VI of the liver. (**F**) Contrast-enhanced CT showing a ring-enhancing metastatic nodule in segment VI located adjacent to the root of the Glisson branch to segment VI (G6). The lesion was deeply situated and appeared to involve the G6 branch.

Colonoscopy revealed a circumferential obstructing tumor in the rectum (**Fig. 1A**). Contrast-enhanced CT revealed rectal cancer with associated bowel obstruction and a 35-mm metastatic lesion in segment VII of the liver (**Fig. 1B** and **1C**). Based on these findings, he was diagnosed with rectal cancer, cT4aN1bM1a, Stage IVA, according to the Union for International Cancer Control (UICC) 8th edition TNM classification system.

He presented with intestinal obstruction due to rectal cancer and underwent colonic stent placement (self-expandable metallic stent). Following the institutional policy of bridge to surgery (BTS), surgical resection was performed after intestinal decompression. He underwent laparoscopic high anterior resection for rectal cancer. Peritoneal invasion was observed; therefore, combined resection, including the involved peritoneum, was performed.

Histopathological examination revealed well-differentiated adenocarcinoma, classified as pT4bN1bM1a, Stage IVA (UICC). Invasion of the parietal peritoneum was observed. Further microscopic findings included vascular invasion, lymphatic invasion, and perineural invasion. Surgical margins were negative, and 2 of 42 regional lymph nodes examined showed metastasis.

Gadoxetic acid-enhanced MRI confirmed the lesion in segment VII identified on CT and additionally revealed 2 smaller hepatic metastases in segment VI (**Fig. 1D**–**1F**). No other distant metastases were identified. Based on these findings, the disease was considered resectable, and hepatectomy was planned.

**Fig. 2:**
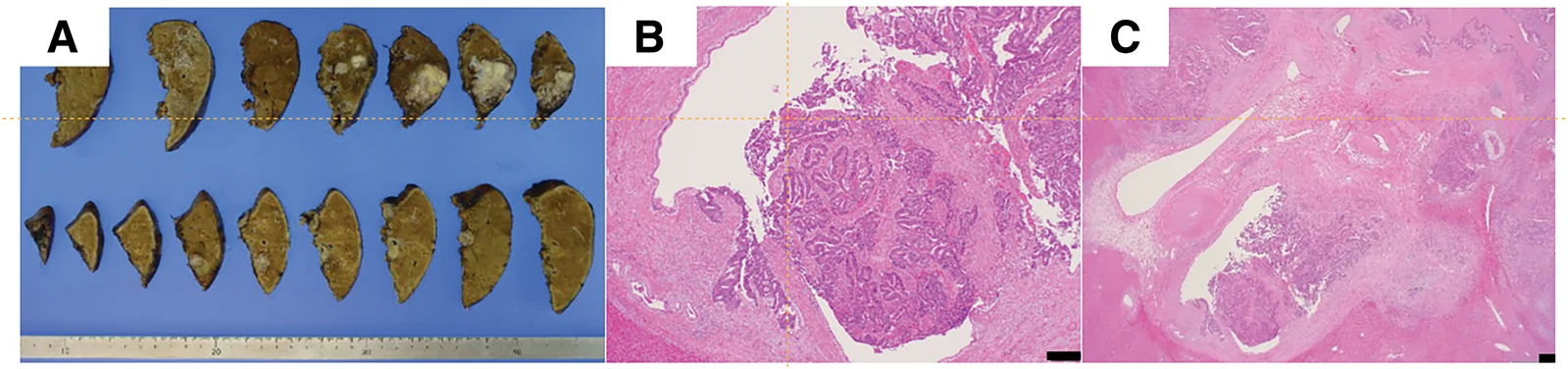
(**A**) Resection margins were negative. (**B**, **C**) Mild intrabiliary extension was observed. Scale bar = 200 μm.

One month after primary tumor resection, he underwent laparoscopic posterior sectionectomy of the liver. Histopathological examination confirmed metastatic rectal adenocarcinoma. Resection margins were negative (**Fig. 2A**). Mild intrabiliary growth (IG) was observed within the resected specimen (**Fig. 2B** and **2C**). No adjuvant therapy was administered.

**Fig. 3:**
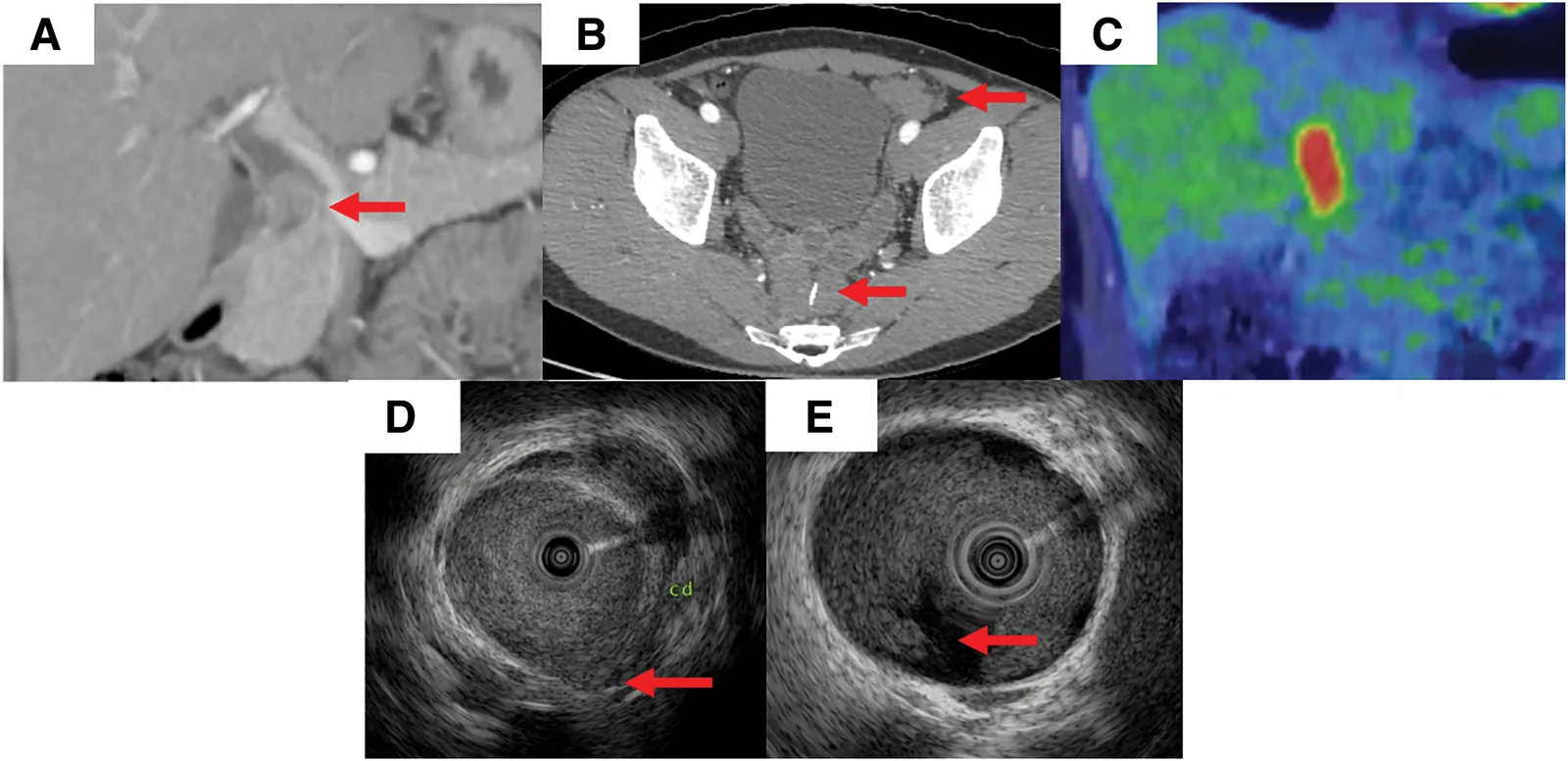
(**A**) Contrast-enhanced CT showing a mass lesion in the common bile duct. (**B**) Contrast-enhanced CT demonstrating local recurrence in the rectum and pelvic recurrence. (**C**) FDG-PET/CT depicting abnormal uptake in the common bile duct (SUVmax 9.1). (**D**) EUS showing an irregular mucosal surface within the common bile duct and a hypoechoic mass filling the lumen. (**E**) EUS of a protruding mass lesion within the common bile duct. EUS, endoscopic ultrasonography

**Fig. 4:**
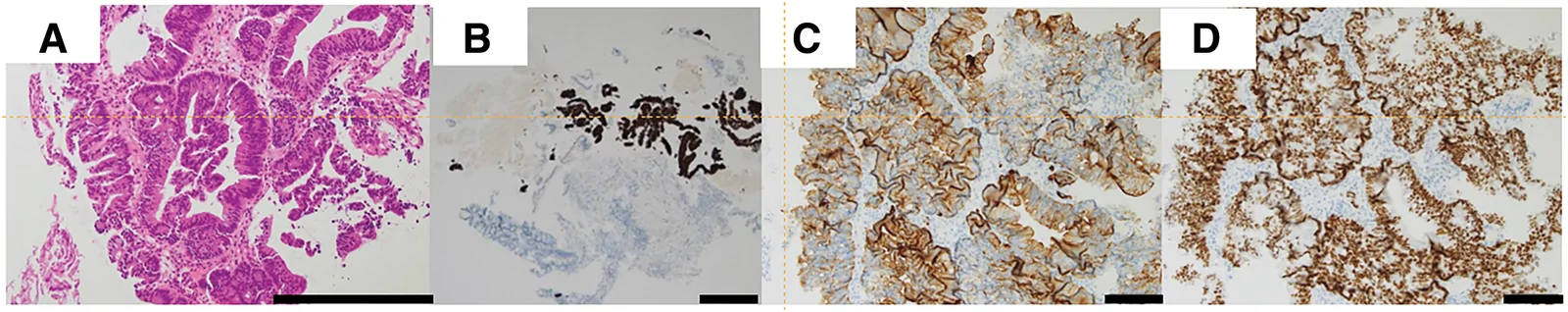
(**A**) HE staining showing adenocarcinoma within the common bile duct epithelium. Immunohistochemical staining revealing CK7 (**B**) negativity and CK20 (**C**)/CDX2 (**D**) positivity, consistent with metastasis from rectal adenocarcinoma. Scale bar = 500 μm. CK7, cytokeratin 7; CK20, cytokeratin 20; HE, hematoxylin and eosin

Eight months after hepatic resection, CT revealed a mass lesion in the common bile duct, and local recurrence in the rectum and pelvic recurrence were also identified (**Fig. 3A** and **3B**). PET-CT showed abnormal fluorodeoxyglucose (FDG) uptake (SUVmax: 9.1) corresponding to the lesion in the common bile duct (**Fig. 3C**). Endoscopic ultrasonography (EUS) revealed an irregular mucosal surface within the common bile duct and a hypoechoic mass filling the lumen (**Fig. 3D**). The lesion appeared as a protruding mass within the bile duct (**Fig. 3E**). Based on these ultrasonographic findings, invasion from adjacent lymph nodes was considered unlikely. A biopsy of the mass lesion in the common bile duct was performed. Histopathological examination with hematoxylin and eosin (HE) staining, along with immunohistochemistry, revealed a cytokeratin (CK) 7-negative, CK20-positive, and caudal-type homeobox 2 (CDX2)-positive profile, consistent with metastasis from rectal adenocarcinoma (**Fig. 4A**–**4D**).

Comprehensive genomic profiling revealed wild-type RAS and BRAF genes, no *HER2* overexpression, and microsatellite-stable (MSS) status. Based on these findings and the patient’s general condition, a regimen of CAPOX plus panitumumab was initiated.

Ten months after hepatic resection, he developed obstructive jaundice due to metastasis to the common bile duct. Endoscopic retrograde cholangiopancreatography (ERCP) was performed, and a plastic biliary stent was placed. He experienced recurrent obstructive jaundice due to stent occlusion, as well as stent migration. Multiple stent exchanges were performed, including conversion to a self-expandable metallic stent and size adjustments, to maintain biliary drainage. Owing to these interventions, biliary obstruction remained well controlled.

He subsequently developed colonic stricture due to local recurrence, and a self-expandable metallic stent was placed, successfully relieving the obstruction.

New hepatic metastases appeared after 8 cycles of the first regimen; therefore, treatment was modified to CAPIRI plus bevacizumab. After 12 cycles of the second regimen, hepatic metastases showed slow progression, and therapy was switched to FOLFIRI plus ramucirumab; he has since completed 5 cycles. He has survived for 24 months since the diagnosis of common bile duct metastasis and remains alive under ongoing follow-up.

## DISCUSSION

EHBD metastasis from colorectal cancer is extremely rare; therefore, there is no established consensus on treatment. Since its first description by Herbut and Watson in 1946,^3)^ only 14 cases have been published in PubMed (keywords: (colorectal OR rectal) AND (“bile duct metastasis” OR “biliary metastasis”)). **Table 1** summarizes 14 cases, including the case presented herein.^2,4–14)^

**Table 1:** Summary of reported cases of EHBD metastasis from colorectal cancer

Case	Author	Year	Age	Sex	Recurrence time to EXBD metastasis (months)	Survival after EHBD metastasis (months)	Tumor site	Treatment	IG in liver metastases	Metastatic pathways	Liver metastases	Perioperative chemotherapy (NAC/Adjuvant)	Chemotherapy after recurrence	Molecular profile
1	Kobayasi	2011	71	M	61	12	Common bile duct	Endoscopic procedure	−	Implantation/lymphatic	+	FOLFIRI (Adjuvant)	Chemotherapy (details not reported)	NA
2	Yamao	2015	65	F	94	NA	Distal bile duct	SSPPD	−	Implantation	+	SOX+Cmab (NAC)	NA	KRAS WT
3	Kawakatsu	2015	73	M	12	12	Intrapancreatic bile duct	SSPPD	+	Implantation	+	None	None	NA
4	Lee	2015	70	M	0	3	Distal bile duct	PPPD	/	NA	−	Chemotherapy (adjuvant; details not reported)	/	NA
5	Strauss	2016	57	M	NA	NA	Common bile duct	Endoscopic stenting	NA	NA	+	FOLFOX+Bev →FOLFIRI+Bev	FOLFIRI+Bev	NA
6	Koh	2017	NA	NA	NA	NA	Distal bile duct	Endoscopic procedure	NA	NA	NA	NA	NA	NA
7	Koh	2017	NA	NA	NA	NA	Confluence of hepatic ducts	Endoscopic procedure	NA	NA	NA	NA	NA	NA
8	Knowles	2017	50	M	19	7	Confluence of hepatic ducts	Endoscopic stenting	NA	NA	NA	NA	KRAS inhibitor (details not reported)	KRAS mutant
9	Ofuchi	2020	69	M	48	NA	Hilar bile duct	Extended right hemihepatectomy	+	Implantation/hematogenous	+	NA	NA	NA
10	Nakagawa	2020	74	F	70	24	Common bile duct	PPPD	+	Implantation/hematogenous/lymphatic	+	None	None	NA
11	Da Silva	2021	65	M	0	/	Primary biliary confluence	Bile drainage	/	NA	−	NA	/	NA
12	Majdoubi	2024	60	F	10	18	Hilar bile duct	Endoscopic stenting	NA	Hematogenous	+	CAPOX (NAC) →CAPOX (adjuvant)	CAPOX	NA
13	Monoe	2025	49	M	19	4	Distal common bile duct	Endoscopic stenting	NA	Implantation/PBCP	+	Recurrence before initiation	FOLFOX+Bev →FOLFIRI+Bev	NA
14	This case	2025	46	M	8	24	Common bile duct	Endoscopic stenting	+	Implantation/hematogenous/lymphatic/PBCP	+	CAPOX+Pan	CAPIRI+Bev → FOLFIRI+Ram	RAS WT

Bev, bevacizumab; EHBD, extrahepatic bile duct; F, female; IG, intrabiliary growth; M, male; NA, not available; Pan, panitumumab; PBCP, peribiliary capillary plexus; PPPD, pylorus-preserving pancreaticoduodenectomy; Ram, ramucirumab; SSPPD, subtotal stomach-preserving pancreaticoduodenectomy

**Table 1** shows only 1 reported case in which a patient achieved long-term survival of at least 2 years after the diagnosis of EHBD metastasis following curative surgical resection. Thus, surgical resection should be considered when there is no evidence of distant metastasis and curative resection is feasible. Similar to our case, Kawakatsu et al.^6)^ suggested that an aggressive surgical approach for recurrence of biliary metastasis from colorectal cancer may improve survival. However, as indicated in **Table 1**, pancreaticoduodenectomy is required for surgical treatment, and patient age, ADL, and prognosis must be carefully considered when determining surgical indications.

In our situation, radical surgery was not possible because of distant metastases, including liver metastasis and local recurrence. However, this case represents a rare and prognostically favorable example of EHBD metastasis from colorectal cancer, achieving the longest reported survival of more than 2 years without surgical resection through combined biliary stenting and systemic chemotherapy. Frequent stent exchanges and size adjustments were required to control biliary obstruction, but the patient survived for more than 2 years after bile duct metastasis while maintaining both survival and QOL.

Stent placement is not curative, but it is essential for managing obstructive jaundice and cholangitis and for enabling continuation of chemotherapy. Nevertheless, stent placement also carries risks, including stent occlusion, migration, cholangitis, and pancreatitis.^15)^ In this young patient, the risk of severe post-ERCP pancreatitis was considered high. Therefore, plastic stenting was initially selected because it is generally associated with a lower incidence of procedure-related pancreatitis and was later upgraded to a self-expandable metallic stent as part of a long-term treatment strategy.

EHBD metastases, particularly distal lesions, are difficult to distinguish from primary cholangiocarcinoma based on imaging or clinical findings alone. The practicality of CK7, CK20, and CDX2 immunostaining in differential diagnosis has been previously recorded in the literature. Rullier et al.^16)^ reported that the CK7(−)/CK20(+) pattern was present in only 4% of bile duct cancers but in 81% of colorectal cancers and that combined CK7 and CK20 staining is useful in determining colorectal origin.^7)^ Furthermore, CDX2 and SATB2 (special AT-rich sequence-binding protein)^2)^ are highly sensitive and specific markers for metastatic lesions of gastrointestinal tract adenocarcinoma, especially colon cancer.^17)^ In our case, the immunostaining profile confirmed colorectal origin, which was critical for accurate diagnosis and treatment planning.

IG is a characteristic growth pattern occasionally observed in colorectal liver metastases, with a reported prevalence of 3.6%–10.6%.^18)^ As IG frequently causes biliary obstruction and ductal dilatation, it may mimic cholangiocarcinoma and result in diagnostic difficulty.^18)^ Estrella et al.^18)^ reported that major duct involvement was associated with obstructive liver dysfunction, radiological biliary abnormalities, and secondary sclerosing cholangitis, but had no significant impact on overall survival.

The metastatic pathways to the EHBD are not fully understood; however, several mechanisms have been proposed, including (1) hematogenous dissemination, (2) lymphatic dissemination, (3) implantation of cancer cells, and (4) direct infiltration through the peribiliary capillary plexus (PBCP).^11)^ Implantation is considered to occur when detached tumor cells adhere to injured biliary epithelium and subsequently proliferate. Shedding of cancer cells can be triggered by external factors, such as operative procedures including endoscopy, surgery, or radiofrequency ablation (RFA),^2)^ or can occur spontaneously. Bile flow may then transport these cells to sites of bile duct mucosal injury. The PBCP, a fine capillary network beneath the biliary epithelium, may also serve as a route for tumor infiltration into the bile duct wall. Through this pathway, intraductal tumors may form without visible mucosal disruption.^14)^

**Table 1** shows that among the 14 patients with EHBD metastasis from colorectal cancer, 7 underwent liver resection. Of these 7 cases, 4 exhibited IG. Among the remaining 3 cases, 1 lacked documentation of IG, and 2 were confirmed to be negative for extension. One of these 2 cases received neoadjuvant chemotherapy, suggesting the possibility that IG might have disappeared as a result of treatment. These findings suggest that IG is frequently observed in cases of colorectal liver metastasis with extrahepatic biliary involvement. In addition, among the 8 reports discussing the metastatic pathway, 7 suggested implantation, while 1 attributed spread to hematogenous dissemination. It is important to note that all reports exhibiting IG mentioned implantation as a potential underlying mechanism.

In our case, the presence of vascular and lymphatic invasion, along with intraductal tumor extension, means that the possibility of all 4 metastatic routes cannot be entirely ruled out.

However, given the observed IG and the fact that hepatectomy had been performed, we considered implantation to be the most probable route of metastasis, consistent with observations from other reported cases. Nevertheless, further accumulation of case reports and data is essential to clarify the precise mechanism of metastasis in these situations.

## CONCLUSIONS

Our report is the fourteenth to describe EHBD metastasis from colorectal cancer. This case showcases one of the longest reported survivals achieved through a multidisciplinary approach among patients with unresectable disease. Endoscopic biliary stent placement for metastatic involvement of the common bile duct represents a minimally invasive and effective modality for palliation of biliary obstruction. However, as demonstrated in the present case, clinically relevant complications such as stent occlusion and migration may occur, underscoring the importance of meticulous long-term surveillance and the establishment of an appropriate strategy for timely re-intervention.

## DECLARATIONS

### Availability of data and materials

The data supporting the conclusions of this article are included within the article.
